# Daytime variation of in-hospital mortality and low cardiac output syndrome after pediatric cardiac surgery-a retrospective cohort study

**DOI:** 10.1080/07853890.2024.2430764

**Published:** 2024-11-22

**Authors:** Chaoyang Tong, Xinwei Du, Kan Zhang, Mengqin Shan, Haibo Zhang, Jijian Zheng

**Affiliations:** a Department of Anesthesiology, Shanghai Children’s Medical Center, School of Medicine and National Children’s Medical Center, Shanghai Jiao Tong University, Shanghai, China; b Department of Pediatric Thoracic and Cardiovascular Surgery, Shanghai Children’s Medical Center, School of Medicine and National Children’s Medical Center, Shanghai Jiao Tong University, Shanghai, China

**Keywords:** Daytime variation, pediatric, cardiac surgery, outcomes

## Abstract

**Objective:**

Recent studies suggest that adult cardiac surgery performed in the morning increases the risk of major adverse cardiac events, but it is unclear whether this association exists in pediatric cardiac surgery. This study aimed to determine whether the composite outcome of in-hospital mortality and low cardiac output syndrome (LCOS) differs between morning and afternoon pediatric cardiac surgeries.

**Methods:**

This retrospective cohort study enrolled 23,433 consecutive pediatric patients who underwent cardiac surgery between August 2014 and December 2021. Pediatric patients who had surgery start time between 8 AM and 11 AM (morning surgery) versus between 2 PM and 5 PM (afternoon surgery) were compared in the risk of the composite outcome. The Society of Thoracic Surgeons-European Association for Cardiothoracic Surgery (STAT) score was used to indicate the surgical complexity. The adjusted odds radio (aOR) for the composite outcome was calculated using multivariate logistic regression. The restricted cubic spline (RCS) was performed to characterize the continuous relationship between the surgery start time and risk of the composite outcome.

**Results:**

Of 16,534 included pediatric patients, 1.2% died after morning surgery and 0.6% died after afternoon surgery. The composite outcome of in-hospital mortality and LCOS occurred in 14.2% (1,507 of 10,591) of morning surgeries and 8.6% (514 of 5,943) of afternoon surgeries: morning versus afternoon aOR, 1.186 (95% CI, 1.046 to 1.344; *p* = 0.008). The association was also determined in children aged 3 to 18 years (aOR = 1.598, *p* = 0.003), weighted between 6.1 to 8.7 kg (aOR = 1.453, *p* = 0.006), or more than 13 kg (aOR = 1.488, *p* = 0.019), and with STAT category 4-5 (aOR = 1.367, *p* = 0.014) subgroups. The RCS plot showed that the aOR of the composite outcome decreased with a delay in the start time of surgery.

**Conclusion:**

Our study supports the selective afternoon scheduling of specific pediatric cardiac surgeries, but further investigation is needed in a multicenter cohort.

**KEY MESSAGES**
- This study reviewed 23,433 consecutive pediatric patients who underwent cardiac surgery between August 2014 and December 2021.
- This study suggested that afternoon surgery was associated with a lower risk of the composite outcomes of in-hospital mortality and low cardiac output syndrome.
- The above association was also determined in children aged 3 to 18 years, weighted between 6.1 and 8.7 kg, or more than 13 kg, and with STAT category 4-5 subgroups.
- The restricted cubic spline plot showed that the adjusted risk of the composite outcome decreased with a delay in the start time of surgery.
- Our findings support selective afternoon scheduling for specific pediatric cardiac surgery.

## Introduction

Circadian rhythm is an intrinsic biological oscillation driven by an endogenous biological clock that plays an important role in regulating various body functions, including the cardiovascular [1–2]. A profound daytime dependence of ischemia-reperfusion tolerance mediated by the cardiomyocyte circadian clock has been demonstrated in experimental animals [3–4]. This tolerance has also been observed in the nonsurgical population, where patients with myocardial ischemia have larger myocardial infarcts, more heart failure, and worse left ventricular dysfunction from midnight to 6 AM than in the afternoon [5–7]. A large prospective First Acute Myocardial Infarction study reported that ST-segment elevation myocardial infarction occurred more frequently between 6 AM and noon [8]. Cardiac surgery provokes a predictable perioperative myocardial ischemia-reperfusion injury that is associated with adverse clinical outcomes [9–12], and it is unclear whether such biorhythm regulation exists in this surgical treatment.

Currently, there is conflicting evidence concerning these issues in cardiac surgery. Montaigne et al. showed that patients receiving aortic valve replacement in the afternoon had improved myocardial outcomes compared to patients who underwent surgery in the morning. The mechanism for reducing myocardial injury may be mediated by regulating the expression of the circadian rhythm gene, *Rev-Erbα* [13]. However, several studies reported no difference in cardiac-related or other complications and in-hospital mortality when cardiac surgery was performed in the morning versus afternoon [14–18] or overnight [19–20]. Some studies have suggested that cardiac surgery occurring later in the day is related to increased morbidity and mortality, probably due to reduced availability of personnel and hospital resources, provider fatigue, loss of attention, and hurriedness [21–23].

In view of the emerging experimental evidence demonstrating age-associated changes in circadian functions [24–25], whether daytime variation in perioperative cardiac events exists in pediatric patients undergoing cardiac surgery is unknown. Thus, based on a large dataset collected prospectively, this study aimed to determine whether the composite outcome of in-hospital mortality and low cardiac output syndrome (LCOS) differs in daytime variation after pediatric cardiac surgery.

## Materials and methods

### Study design and patients

This study was a secondary analysis of dataset from a previously published article that evaluated the performance of five machine learning (ML) algorithms for predicting four major APOs after pediatric congenital heart surgery [26]. Compared to the above-mentioned published articles, our current manuscript mainly focused on the impact of morning versus afternoon surgery on cardiac composite outcomes. Also, in the selection of the composite outcomes, we added deaths recorded in the hospital electronic medical records to maintain consistency with the composite outcomes of previous studies.

**Figure 1.**
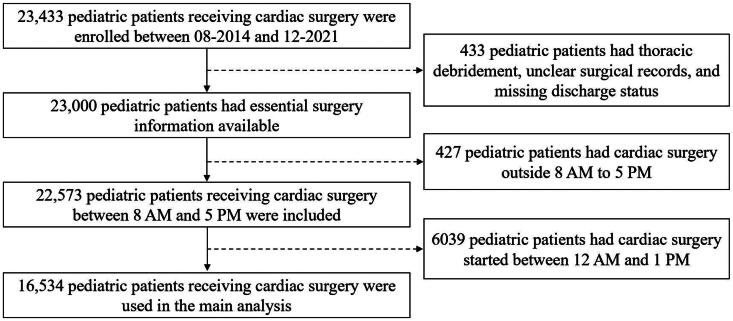
Patient flow chart.

Following approval from the Institutional Review Board (IRB) of Shanghai Children’s Medical Center (Permission NO: SCMCIRB-K2022194; Date: 12 December 2022), we performed a single-center retrospective cohort study including 23,433 consecutive pediatric patients who received cardiac surgery between August 2014 and December 2021. Patients who underwent thoracic debridement, those with unclear surgical records, those with missing discharge status, and those with surgery start time outside 8 AM to 5 PM were excluded. Patients with surgery start time between 12 AM and 1 PM were not included in the primary analysis to provide a clear separation between the two groups. Thus, 16,534 pediatric patients who underwent cardiac surgery were enrolled in the final analysis (Figure 1). The data were anonymized, and the need for informed consent was waived. This study adhered to the STROBE guidelines [27].

### Data collection and study outcome

Perioperative data were prospectively recorded by dedicated clinicians, consistent with the records and definitions in the Society of Thoracic Surgeons (STS) Congenital Heart Surgery Database Collection Form. All patients were followed-up until hospital discharge. This study extracted and pooled existing clinical data from the electronic medical dataset, including patients’ baseline and intraoperative characteristics and cardiac-related complications regarding in-hospital mortality and LCOS. The primary outcome was the composite of in-hospital mortality and LCOS. This clinically relevant outcome for LCOS was chosen because it is believed to result from a combination of underlying heart disease, myocardial ischemia from aortic cross-clamping, residual effects of cardioplegia, and activation of inflammatory pathways caused by blood exposure to foreign surfaces during cardiopulmonary bypass (CPB) [28–29].

### Definition

In-hospital mortality was defined as death during the same hospital stay as surgery, regardless of the cause [30]. The definition of LCOS was based on previous literature [28,31]. Specifically, the requirement for two or more inotropic agents with one or more of the following parameters: (1) clinical signs or symptoms of cardiac index <2.2 L/(min·m^2^); (2) decreased central venous oxygen saturation; (3) increased difference in arterial to central venous oxygen saturation; (4) elevated or rapid increase in blood lactate; (5) urine output <1 ml/(kg·h); and (6) increased peripheral skin temperature to core body temperature (>5 °C) lasting for 2 h. Based on previous studies [13,16,17], patients with surgery start time between 8 AM and 11 AM were defined as developing morning surgery, and those with surgery start time between 2 PM and 5 PM were defined as developing afternoon surgery. A total of 31 physicians qualified as chief surgeons in charge of the 31 groups who performed all surgical procedures. Surgical experience was categorized into two groups (less experience <730 cases and more experience ≥730 cases) based on an average of 728 cases per surgeon (Supplementary Figure 1).

### Subgroup analysis

This study performed subgroup analyses to determine whether the interaction between morning and afternoon surgery in the risk of the composite of in-hospital mortality and LCOS differed by age, weight, and STS-European Association for Cardiothoracic Surgery (STAT) score. Pediatric patients were classified into four groups (< 29 days, 29 days to 1 year, 1–3 years, and 3–18 years). Based on the interquartile range of all data, this study divided weight recorded on the operation day into the following four groups: ≤ 6.1 kg, 6.1 to 8.7 kg, 8.7 to 13.0 kg, and >13 kg. Given the diversity of anatomic diagnoses and varying types of pediatric cardiac surgery, this study used a STAT score that could accurately predict the risk of in-hospital mortality, indirectly indicating surgical complexity [30]. Besides, in a prior sensitivity analysis, we treated surgery start time as a continuous exposure, including 22,573 pediatric patients who underwent cardiac surgery between 8 AM and 5 PM, and evaluated the association between surgery start time and the risk of the composite outcome using a logistic regression model with confounding factors directly adjusted.

### Statistical analysis

Statistical power calculations were not conducted prior to this study, because the sample size was based on the data available in our dataset. Statistics and data analysis plans were formulated before accessing the dataset and completed after the data were accessed. Continuous variables were compared using two independent sample t-tests or Mann-Whitney U tests based on the timing of surgery. Categorical variables were compared using the chi-square test or Fisher’s exact test depending on the sample size. Univariate analysis showed that all variables that were significantly correlated with the composite outcome were included in the multivariate logistic regression model. Restricted cubic spline (RCS) functions that permitted nonlinear associations can be used to assess the inflection point at which the risk of a certain outcome changes; put simply, these functions allow researchers to circumvent the trap of arbitrary dichotomous continuous variables [32–33]. In this study, we used RCS to characterize the continuous relationship between surgery start time and the risk of the composite outcome, treating 9 AM as the reference, with adjustment for all factors. SPSS version 25.0 statistical software (IBM Corp, Armonk, NY, USA) was used for data processing. R version 4.1.2 was performed using the rms and ggplot2 packages. *P*-value <0.05 was considered statistically significant.

## Results

### Study cohort

The baseline and intraoperative characteristics between the morning and afternoon surgeries are presented in Table 1. Of the 16,534 pediatric patients included, 10,591 (64.1%) underwent cardiac surgery in the morning. The morning surgery group was mostly male (54.7% vs. 52.2%, *p* = 0.002), with a younger age (median [Q1,Q3], 10.7[4.9–31.8] vs. 13.4[6.0–34.5], *p* = 0.012), a shorter height (79.4 ± 23.8 vs. 81.0 ± 22.5, *p* < 0.001), and weight (10.8 ± 8.3 vs. 11.1 ± 7.5, *p* = 0.026), and had lower rates of premedication (31.1% vs. 37.5%, *p* < 0.001), respiratory disease (3.5% vs. 4.2%, *p* = 0.018), and mechanical ventilation (1.6% vs. 2.2%, *p* = 0.004). Additionally, morning surgery group had a greater preoperative hematocrit (35.4 ± 6.1 vs. 34.7 ± 5.2, *p* < 0.001) and STAT scores (0.3[0.2–0.8] vs. 0.2[0.2–0.6], *p* < 0.001), longer operative time (164.7 ± 76.8 vs. 138.9 ± 61.0 mins, *p* < 0.001) and anesthesia time (208.2 ± 81.0 vs. 174.1 ± 64.2 mins, *p* < 0.001), higher rates of American Society of Anesthesiologists grade (for II-III, 90.7% vs. 88.4%, *p* < 0.001), of oxygen support (17.0% vs. 9.5%, *p* < 0.001), of previous cardiac operation (12.6% vs. 5.1%, *p* < 0.001), of elective surgery (99.6% vs. 97.6%, *p* < 0.001), of CPB (94.2% vs. 91.3%, *p* < 0.001), and higher surgical experience (72.9% vs. 64.3%, *p* < 0.001) (Table 1).

**Table 1.** Baseline and intraoperative characteristics.

	Morning surgery	Afternoon surgery	
Variables^a^	(*n* = 10591)	(*n* = 5943)	*P* Value
Age, months	10.7 (4.9–31.8)	13.4 (6.0–34.5)	0.012^b^
Male sex	5792 (54.7)	3101 (52.2)	0.002^b^
Height, cm	79.4 ± 23.8	81.0 ± 22.5	<0.001^b^
Weight, kg	10.8 ± 8.3	11.1 ± 7.5	0.026^b^
ASA grade			<0.001^b^
I-II	981 (9.3)	687 (11.6)	
III-V	9610 (90.7)	5256 (88.4)	
Premedication	3294 (31.1)	2228 (37.5)	<0.001^b^
Preoperative hematocrit	35.4 ± 6.1	34.7 ± 5.2	<0.001^b^
Respiratory disease	372 (3.5)	252 (4.2)	0.018^b^
Inotropic support	161 (1.5)	95 (1.6)	0.695
Oxygen support	1802 (17.0)	565 (9.5)	<0.001^b^
Mechanical ventilation	170 (1.6)	133 (2.2)	0.004^b^
Previous cardiac operation	1335 (12.6)	306 (5.1)	<0.001^b^
Elective surgery	10552 (99.6)	5802 (97.6)	<0.001^b^
STAT scores	0.3 (0.2–0.8)	0.2 (0.2–0.6)	<0.001^b^
STAT categories			<0.001^b^
1	5578 (52.7)	3844 (64.7)	
2	2108 (19.9)	1205 (20.3)	
3	1047 (9.9)	313 (5.3)	
4	1803 (17.0)	571 (9.6)	
5	55 (0.5)	10 (0.2)	
Operative time, mins	164.7 ± 76.8	138.9 ± 61.0	<0.001^b^
Anesthesia time, mins	208.2 ± 81.0	174.1 ± 64.2	<0.001^b^
Cardiopulmonary bypass	9977 (94.2)	5428 (91.3)	<0.001^b^
Surgical experience			<0.001^b^
Lower	2866 (27.1)	2119 (35.7)	
Higher	7725 (72.9)	3824 (64.3)	
Year of surgery			<0.001^b^
2014	555 (5.2)	290 (4.9)	
2015	1518 (14.3)	803 (13.5)	
2016	1487 (14.0)	703 (11.8)	
2017	1539 (14.5)	823 (13.8)	
2018	1520 (14.4)	808 (13.6)	
2019	1404 (13.3)	932 (15.7)	
2020	1335 (12.6)	831 (14.0)	
2021	1233 (11.6)	753 (12.7)	

^a^ Continuous data was shown as mean ± standard deviation (SD) or median (interquartile range, Q1, Q3), and categorical data as number (%).

^b^ Statistically significant (*p* < 0.05). ASA, American Society of Anesthesiologists; STAT, Society of Thoracic Surgeons-European Association for Cardiothoracic Surgery.

### Study outcome

The overall incidence of in-hospital mortality was 1.0% (163 of 16,534), 1.2% (129 of 10,591) for morning surgery, and 0.6% (34 of 5,943) for afternoon surgery (morning vs. afternoon unadjusted odds ratio (OR), 2.143; 95% CI, 1.467 to 3.131; *p* < 0.001). The incidence of LCOS was 12.1% (1,996 of 16,534) overall, 14.0% (1,486 of 10,591) for morning surgery, and 8.6% (510 of 5,943) for afternoon surgery (morning vs. afternoon unadjusted OR, 1.739; 95% CI, 1.564 to 1.933; *p* < 0.001). The overall incidence of the composite outcome of in-hospital mortality and LCOS was 12.2% (2,021 of 16,534), 14.2% (1,507 of 10,591) for morning surgery, and 8.6% (514 of 5,943) for afternoon surgery (morning vs. afternoon unadjusted OR, 1.752; 95% CI, 1.577 to 1.947; *p* < 0.001). After adjusting for all confounders, the incidence of the composite outcome still differed significantly between morning and afternoon surgeries, with an estimated OR of 1.186 (95% CI, 1.046 to 1.344; *p* = 0.008).

### Subgroup analysis

The interaction between morning and afternoon surgery in the unadjusted risk of the composite outcome of in-hospital mortality and LCOS differed by age, weight, and STAT scores (Supplementary Figure 2A–C). Overall, the adjusted OR (aOR) of the composite outcome was higher in the morning vs. afternoon surgery, increased with age and STAT score, and decreased with weight regardless of whether surgery was performed in the morning or afternoon (Figure 2A–C). The above association was also determined in children aged 3 to 18 years (aOR = 1.598, 95% CI,1.167 to 2.189, *p* = 0.003), weighted between 6.1 to 8.7 kg (aOR = 1.453, 95% CI,1.113 to 1.898, *p* = 0.006), or more than 13 kg (aOR = 1.488, 95% CI,1.068 to 2.074, *p* = 0.019), and with STAT category 4-5 (aOR = 1.367, 95% CI,1.065 to 1.754, *p* = 0.014) subgroups (Figure 3A–C).

Figure 2. (A) The interaction between morning versus afternoon surgery in the adjusted risk of the composite of in-hospital mortality and LCOS differed by age. (B) The interaction between morning versus afternoon surgery in the adjusted risk of the composite of in-hospital mortality and LCOS differed by weight. **(**C) The interaction between morning versus afternoon surgery in the adjusted risk of the composite of in-hospital mortality and LCOS differed by STAT scores.

**Figure:**
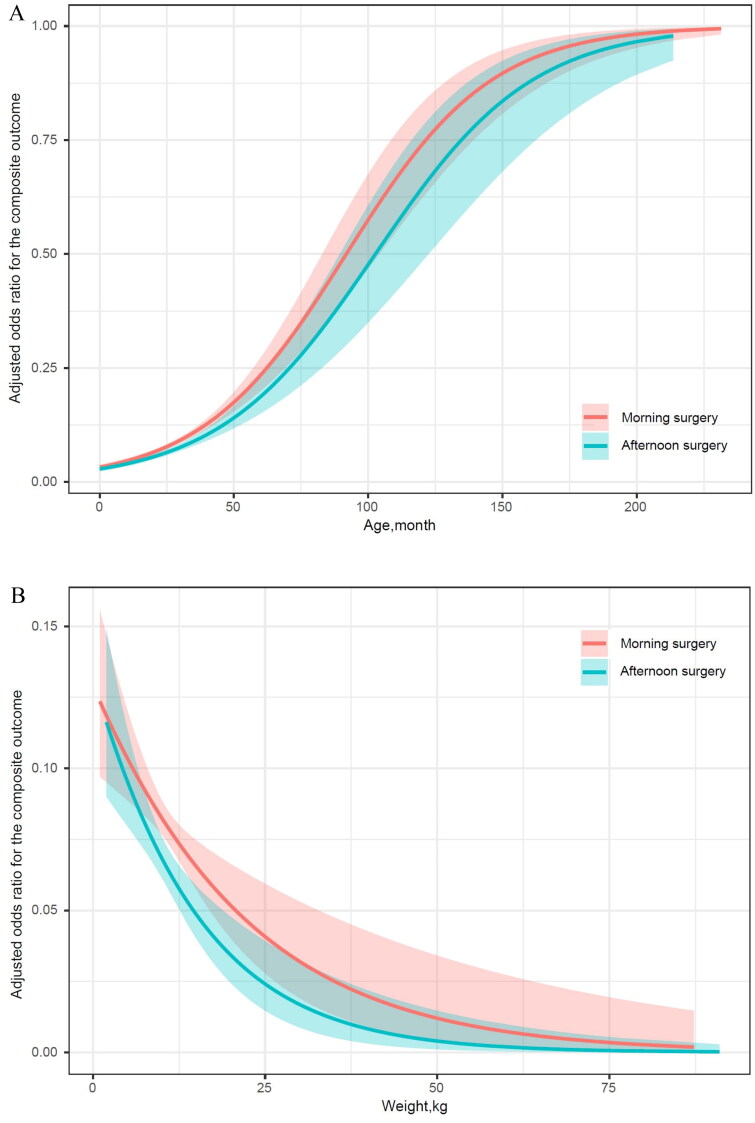

**Figure:**
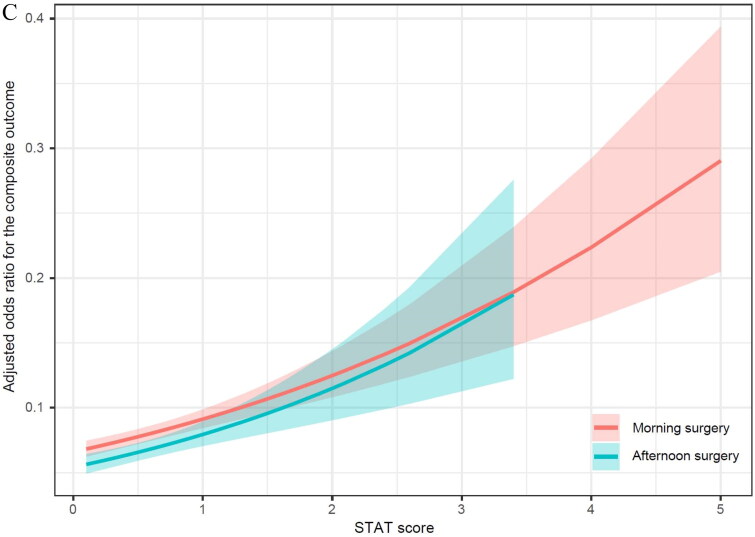

Figure 3. (A) The interaction between morning versus afternoon surgery in the adjusted risk of the composite of in-hospital mortality and LCOS differed by age. (B) The interaction between morning versus afternoon surgery in the adjusted risk of the composite of in-hospital mortality and LCOS differed by weight. (C) The interaction between morning versus afternoon surgery in the adjusted risk of the composite of in-hospital mortality and LCOS differed by STAT scores

**Figure:**
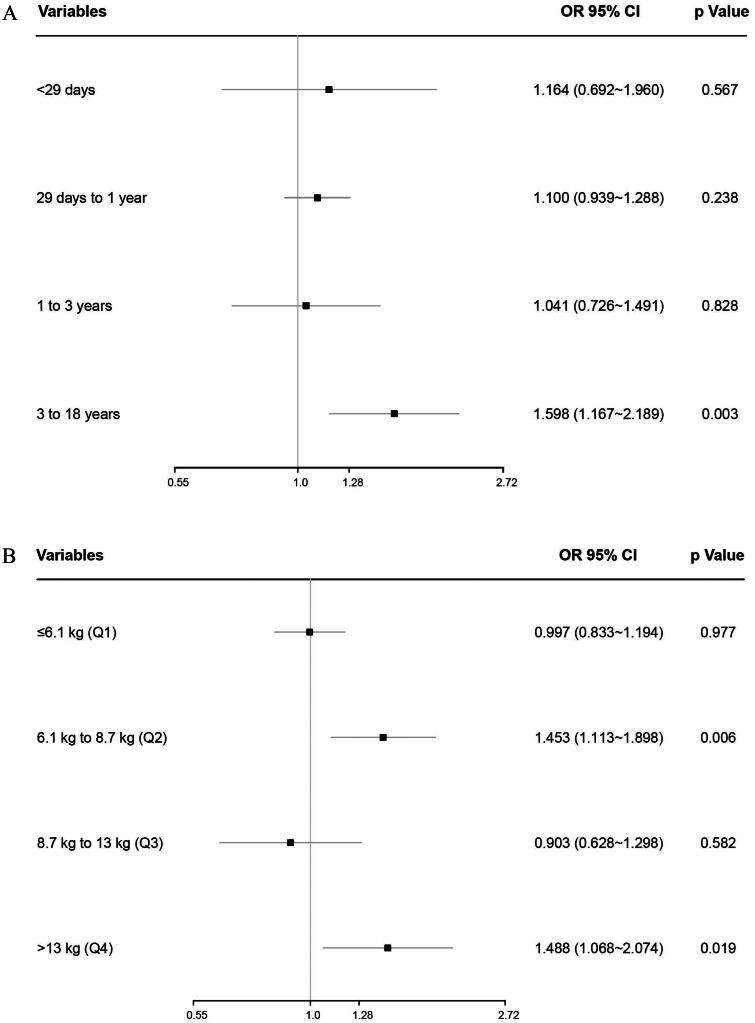

**Figure:**
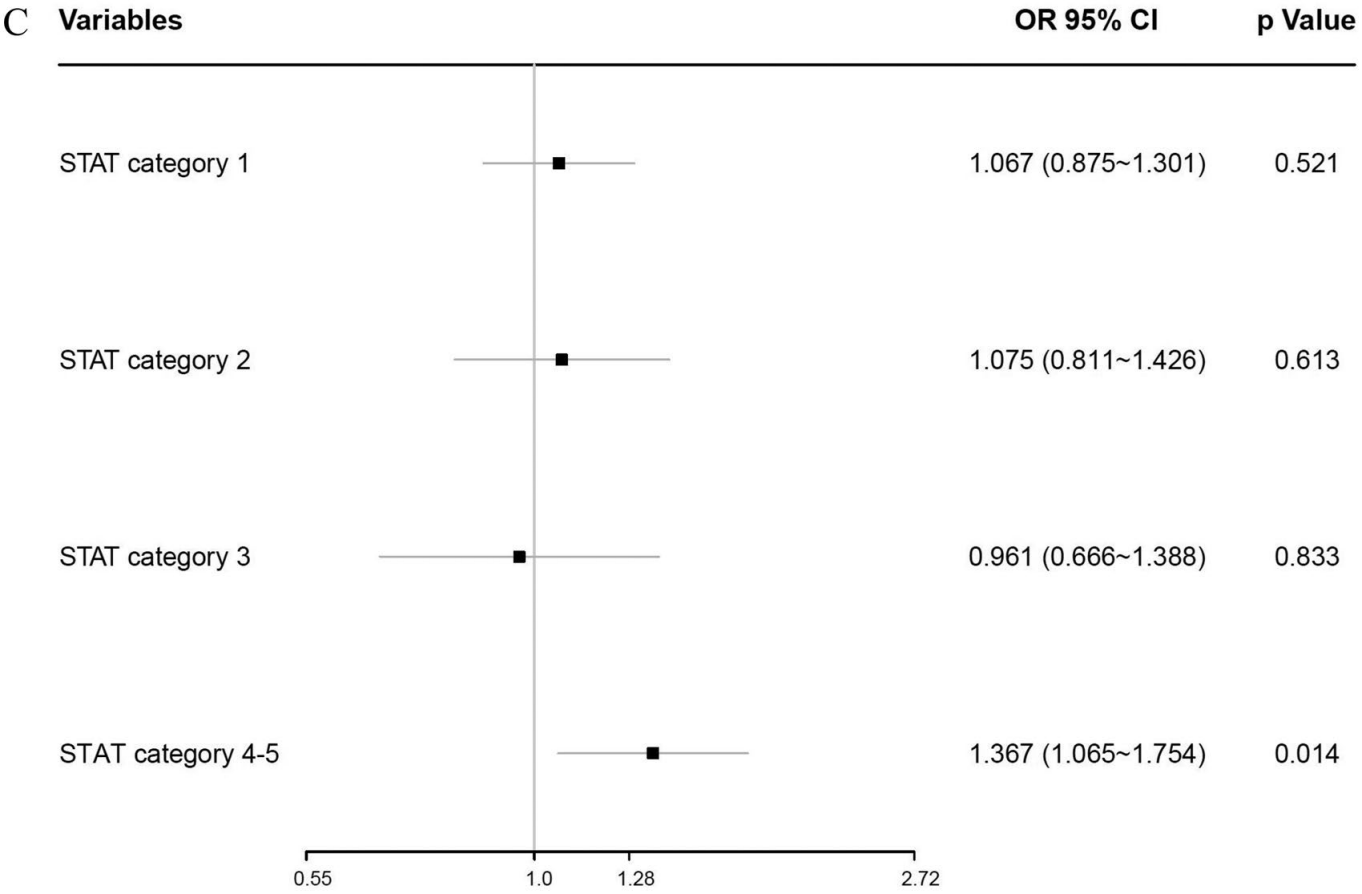

### Post hoc sensitivity analysis

We included 22,573 pediatric patients whose surgeries started between 8 AM and 5 PM. The incidence of the composite outcome varied significantly, from 7.8% at 4 PM, 7.9% at 3 PM, 14.3% at 10 AM, and 14.8% at 9 AM (overall *p* < 0.001; Figure 4A). The RCS plot showed that the aOR of the composite outcome decreased with a delay in the start time of surgery (Figure 4B). The aOR of the composite outcome was 0.819 (95% CI, 0.646 to 1.039, *p* = 0.100), 0.679 (95% CI, 0.480 to 0.962, *p* = 0.030), 0.947 (95% CI, 0.691 to 1.298, *p* = 0.734), 0.846 (95% CI, 0.655 to 1.093, *p* = 0.201), 0.745 (95% CI, 0.573 to 0.970, *p* = 0.028), 0.773 (95% CI, 0.584 to 1.024, *p* = 0.072), 0.667 (95% CI, 0.497 to 0.895, *p* = 0.007), 0.682 (95% CI, 0.494 to 0.942, *p* = 0.020), and 0.740 (95% CI, 0.512 to 1.068, *p* = 0.107) for surgery start time at 9 AM, 10 AM, 11 AM, 12 AM, 1 PM, 2 PM, 3 MP, 4 PM and 5 PM compared to 8 AM, respectively (Figure 4C).

Figure 4. (A) Sensitivity analysis by surgery start time from 8 AM to 5 PM showing the incidence of composite of in-hospital mortality and low cardiac output syndrome. (B) Nonlinear association between surgery start time and the adjusted risk of the composite outcome. (C) The adjusted risk of the composite of in-hospital mortality and low cardiac output syndrome, was compared between pediatric patients by surgery start time subgroups. The adjusted odds ratios (aOR) were reported for other surgery start time versus 8 AM (reference).

**Figure:**
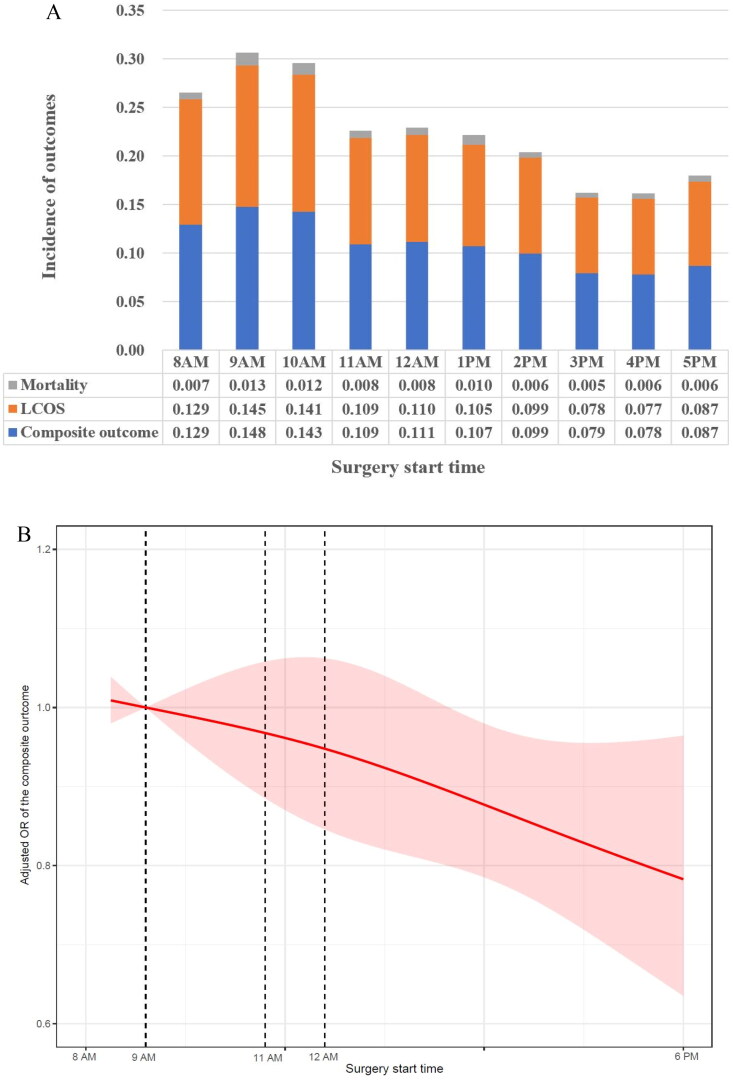

**Figure:**
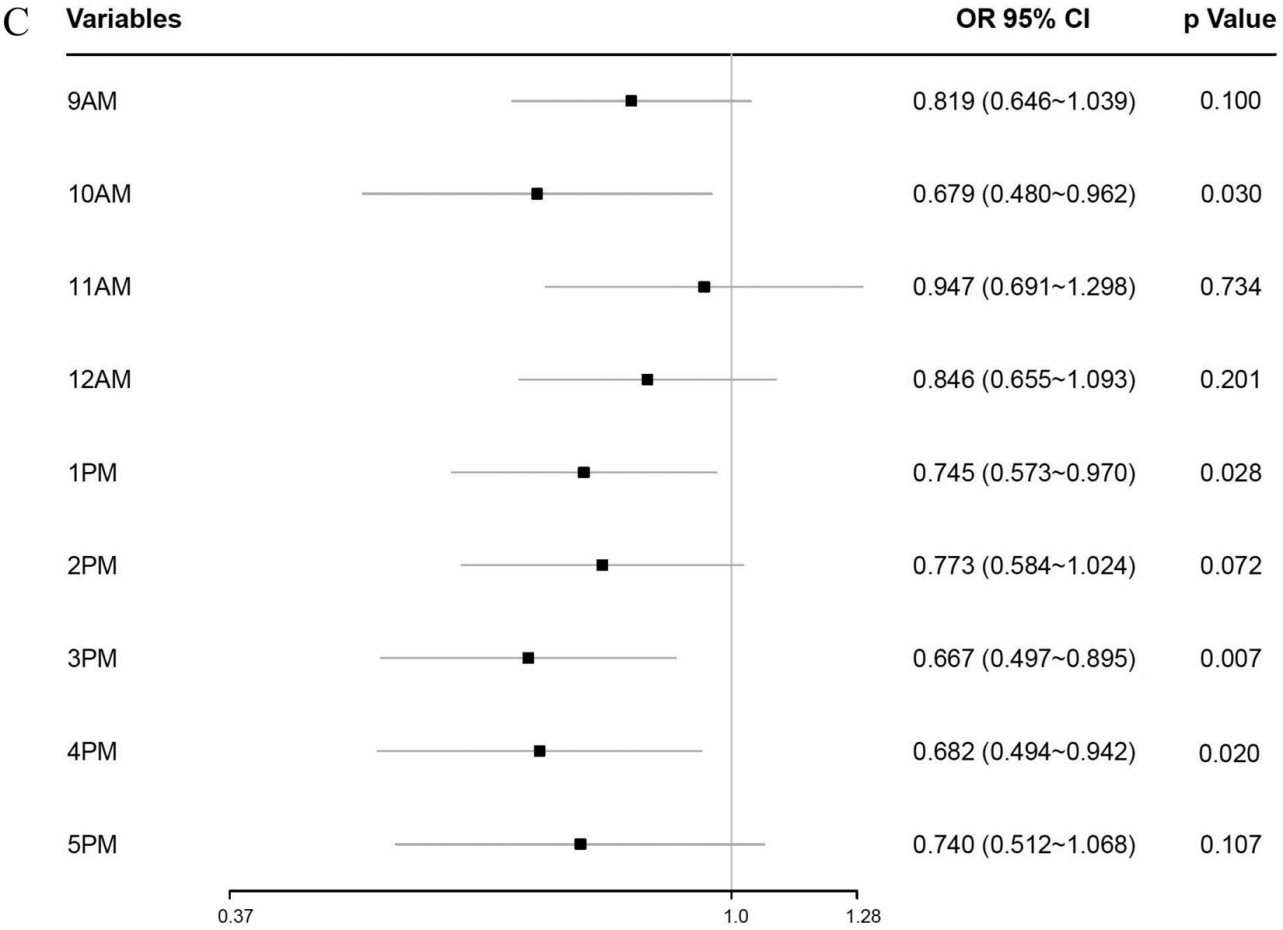

## Discussion

Pediatric patients who had cardiac surgery during the morning developed a higher risk of the composite outcome of in-hospital mortality and LCOS than during the afternoon. The above association was also determined in children aged 3 to 18 years, weighted between 6.1 to 8.7 kg, or more than 13 kg, and with STAT category 4-5 subgroups. The RCS plot showed that the adjusted risk of the composite outcome decreased with a delay in the start time of surgery.

Although inconsistent results of daytime variation on major adverse cardiac events have been observed in adult cardiac surgery [13–23], to the best of our knowledge, this study is the first to investigate the association between daytime variation and pediatric cardiac surgery. This study found that afternoon surgery was associated with a lower incidence of in-hospital mortality and LCOS. This study also determined partial interactions between daytime variation and age, weight, and STAT score, suggesting that such variations may exist only for specific age and weight subgroups, and surgical types. Importantly, the continuous relationship between surgery start time and risk of the composite outcome has not been extensively evaluated, and this study used the RCS plot depicting the gradual reduction in risk of the composite outcome with the delay in the start time of surgery.

The existence of daytime variation in postoperative cardiac composite outcomes in our study was consistent with a previous study by Montaigne et al. who reported a lower incidence of major adverse cardiac events during the afternoon in 298 matched pairs undergoing elective aortic valve replacement surgery with or without coronary artery bypass grafting (CABG) surgery [13]. Conversely, several studies have suggested that daytime variation had no effect on cardiac-related events and mortality, regardless of surgical type and surgery start timing, although the sample sizes were smaller [14–17].

To date, no studies have explored the interaction between daytime variation in cardiac-related events and different age groups, especially in children [13–23]. Surprisingly, the risk of the composite outcome decreased with age regardless of morning or afternoon surgery, while this relationship reversed after adjusting for all confounding factors, showing that younger patients may not be associated with a higher risk of the composite outcome. This study also found that, for children aged 3 to 18 years, selecting afternoon scheduling for cardiac surgery was associated with a lower incidence of composite outcomes. However, such an association was not observed in the other age subgroups. Animal experiments have demonstrated the important role of age in circadian regulation [24–25], and the effect of circadian biology on cardiac adverse events did not differ significantly in children from birth to 3 years, potentially because it was not fully developed. These findings have important implications for selecting patients of a specific age for afternoon surgeries.

The interaction between daytime variation in cardiac-related events and weight has not been widely studied in published literatures [14–17]. Our study showed that lower weight was an independent risk factor affecting the occurrence of composite outcomes. The above association persisted after adjusting for biased factors, namely, the risk of the composite outcome decreased with weight, regardless of whether surgery was performed in the morning or afternoon. Since the effect of age on the composite outcome was reversed after adjusting for all factors, we speculated that weight may be a more important factor in the risk of the composite outcome, which should be of concern to pediatric cardiac surgeons. Furthermore, in a subgroup analysis, this study found that children who weighed 6.1 to 8.7 kg (aOR = 1.453) or more than 13 kg (aOR = 1.488) during morning surgery had a higher risk of composite outcomes. Thus, for children of different weights undergoing cardiac surgery, it should be individualized as far as possible to benefit patients.

Contrary to some published studies showing that different types of surgery performed in the morning or afternoon had no impact on the composite of cardiac-related events [14–17], this study determined the interaction between daytime variation and the STAT score. Subgroup analysis indicated that pediatric patients with preoperative risk stratification identified as STAT category 4-5 selected for afternoon surgery, may benefit from fewer cardiac adverse events. The possible reasons for the difference in the above results were the different incidence of LCOS after adult and pediatric cardiac surgery and the sample size analyzed [17,31]. Besides, myocardial infarction is often considered one of the composite outcomes after adult cardiac surgery, whereas it does not occur after pediatric cardiac surgery, and this term is only defined for patients undergoing CABG [34].

Another important finding of this study is the potential benefit for pediatric patients who underwent cardiac surgery in the afternoon, especially those whose surgery start time was between 3 PM and 4 PM. Correspondingly, the results shown in Figure 4A demonstrate the incidence of the composite outcome from 8 AM to 5 PM, and an increased rate of the composite outcome was observed when surgery started at 9 AM and 10 AM. The RCS plot also confirmed these results and found that the adjusted risk of the composite outcome was negatively correlated with the time to start surgery. Similarly, some previous studies showed that starting elective adult cardiac surgery after 3 or 4 PM does not affect patient morbidity, mortality, or hospital costs [19,21]. Based on the above results, it is conceivable that the combined selection of surgical type and timing of surgery may reduce the risk of cardiac-related adverse events in age-weight-specific patients.

## Limitations

This study had several limitations. First, as a monocentric retrospective cohort study based on a prospectively collected dataset, it has inherent design bias. Second, although the definition of LCOS was based on previously published literature and our report, there may be some bias in the definition given the variety of definitions of LCOS and several clinical indicators included [27,31]. Besides, due to the limited granularity of perioperative care data, detailed arrhythmias were poorly documented, and were not included in the composite outcome because it is a less severe complication than in-hospital mortality and LCOS [35]. Third, other factors that may affect the risk of the composite outcome, such as availability of personnel and hospital resources and provider fatigue, were also not included in this study [21,23]. Finally, this study was restricted to in-hospital adverse events, as we only followed-up until discharge and lacked long-term outcomes after discharge.

## Conclusions

By reviewing the records of 16,534 pediatric patients who underwent cardiac surgery, this study demonstrated that afternoon surgery was associated with a lower risk of the composite outcomes of in-hospital mortality and LCOS. There was an approximately negative continuous relationship between the start time of surgery and the risk of the composite outcome. Our study supports the selective afternoon scheduling of specific pediatric cardiac surgeries, but further investigation and validation are needed in a multicenter cohort.

## Supplementary Material

Supplemental Material

## Data Availability

Our research team could provide original data under reasonable request and with permission from Shanghai Children’s Medical Center.

## Unique identifying number or registration ID

Researchregistry9148.
