# Supplementary material for: Daytime variation of in-hospital mortality and low cardiac output syndrome after pediatric cardiac surgery-a retrospective cohort study
Source: Ann Med. 2024 Nov 22;56(1):2430764. doi: 10.1080/07853890.2024.2430764 (PMC11587732; doi:10.1080/07853890.2024.2430764)
Supplement: Supplemental Material [file IANN_A_2430764_SM8262.zip › suppl_data/Supplementary_materials-legends.docx]

Supplementary Figure 1 Operation volume by 31 surgeon groups

Supplementary Figure 2A: The interaction between morning versus afternoon surgery in the unadjusted risk of the composite of in-hospital mortality and LCOS differed by age. The shadow represents 95% CI of adjusted OR.

Supplementary Figure 2B: The interaction between morning versus afternoon surgery in the unadjusted risk of the composite of in-hospital mortality and LCOS differed by weight. The shadow represents 95% CI of adjusted OR.

Supplementary Figure 2C: The interaction between morning versus afternoon surgery in the unadjusted risk of the composite of in-hospital mortality and LCOS differed by STAT score. The shadow represents 95% CI of adjusted OR.
