# Supplementary figures and images for: Daytime variation of in-hospital mortality and low cardiac output syndrome after pediatric cardiac surgery-a retrospective cohort study
Source: Ann Med. 2024 Nov 22;56(1):2430764. doi: 10.1080/07853890.2024.2430764 (PMC11587732; doi:10.1080/07853890.2024.2430764)

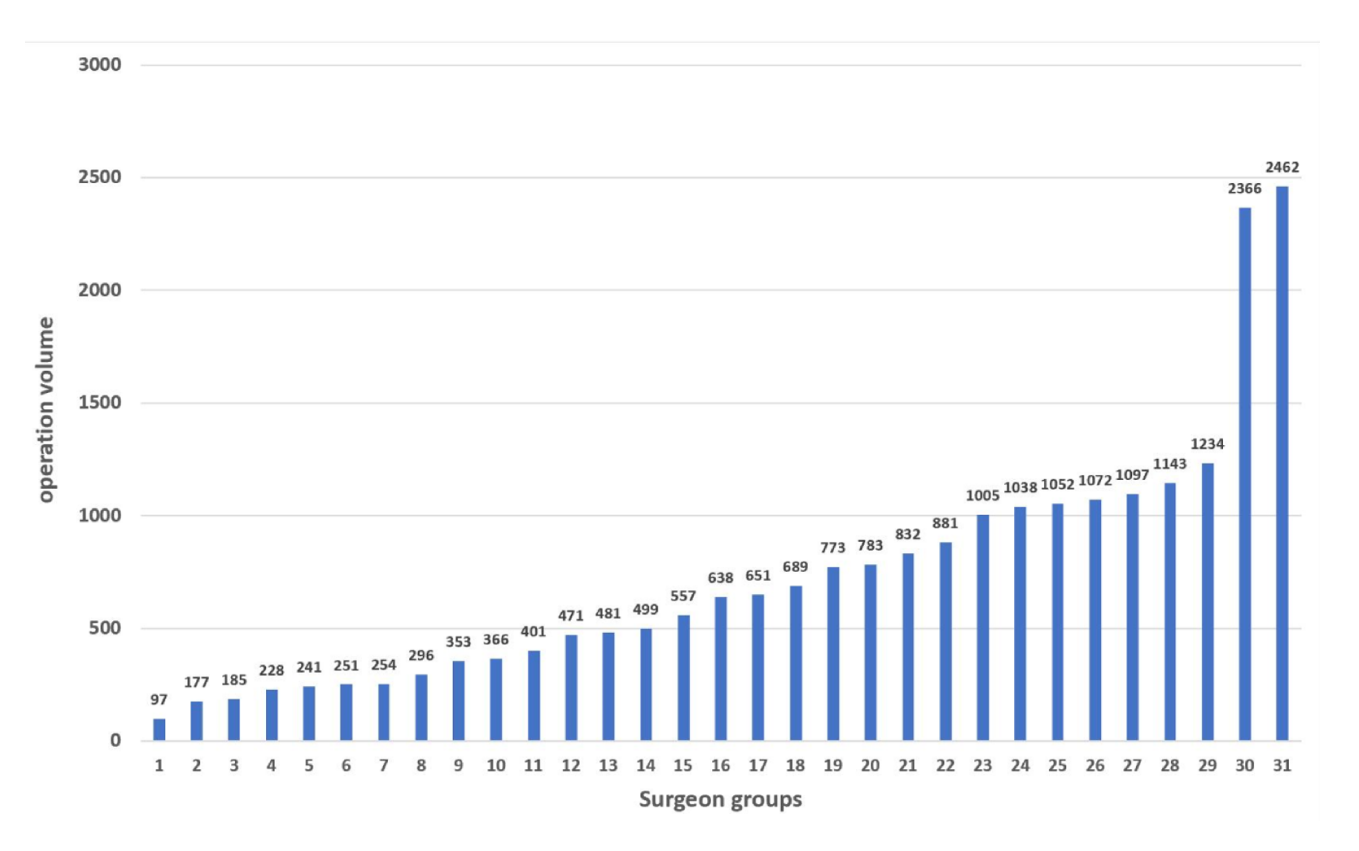

Supplement: Supplemental Material [file IANN_A_2430764_SM8262.zip › suppl_data/Supplementary Figure 1.tif]

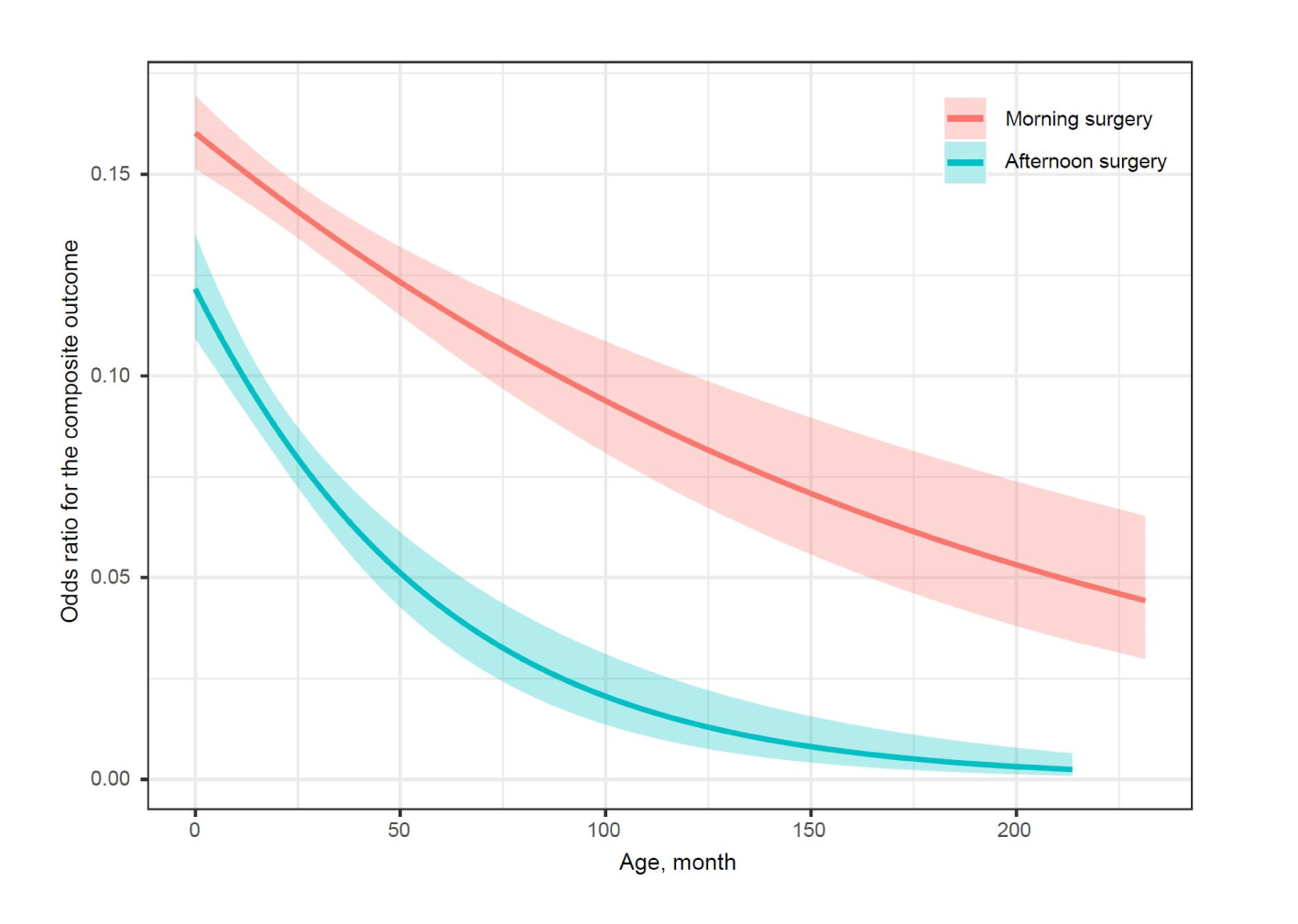

Supplement: Supplemental Material [file IANN_A_2430764_SM8262.zip › suppl_data/Supplementary Figure 2A.tif]

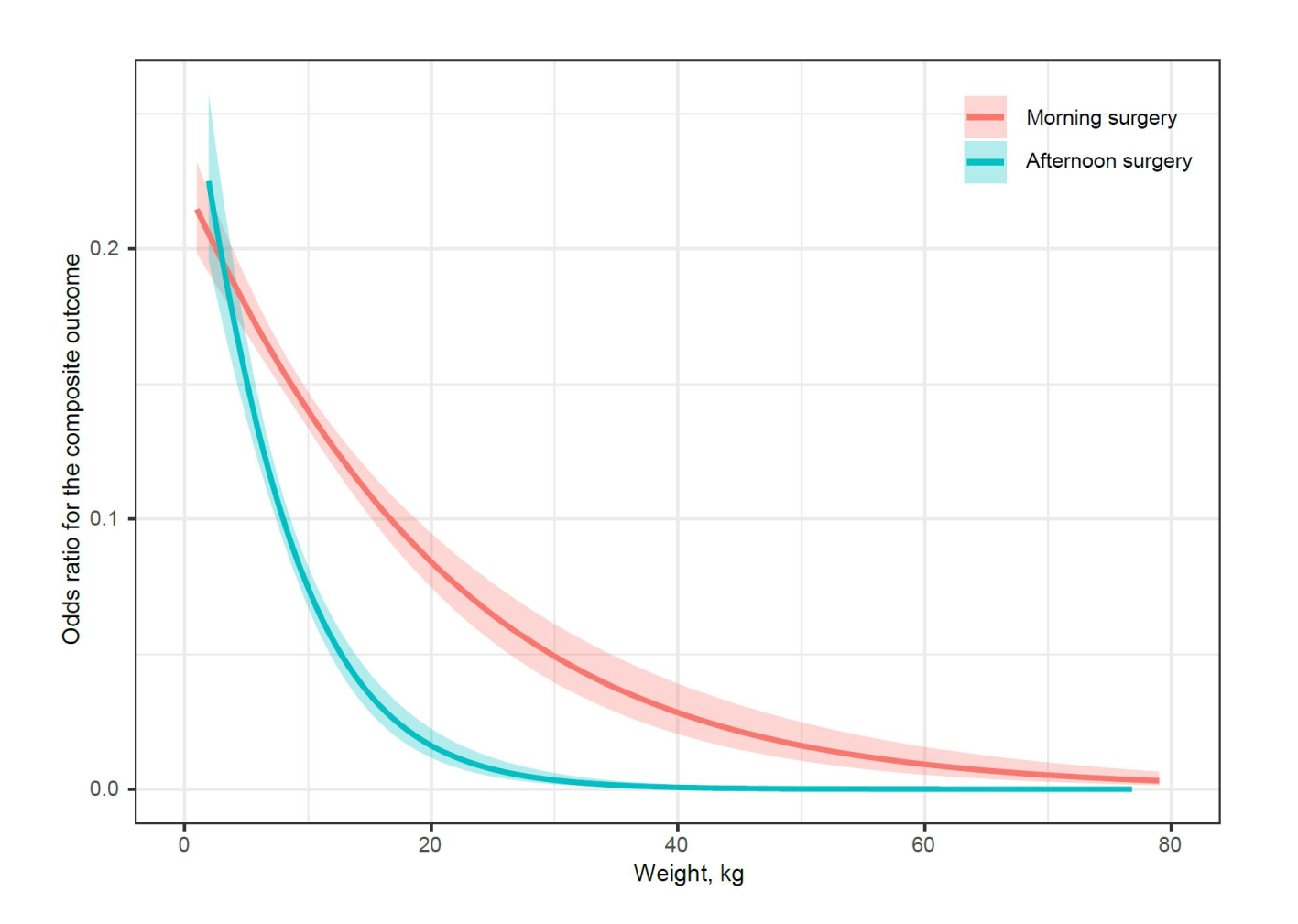

Supplement: Supplemental Material [file IANN_A_2430764_SM8262.zip › suppl_data/Supplementary Figure 2B.tif]

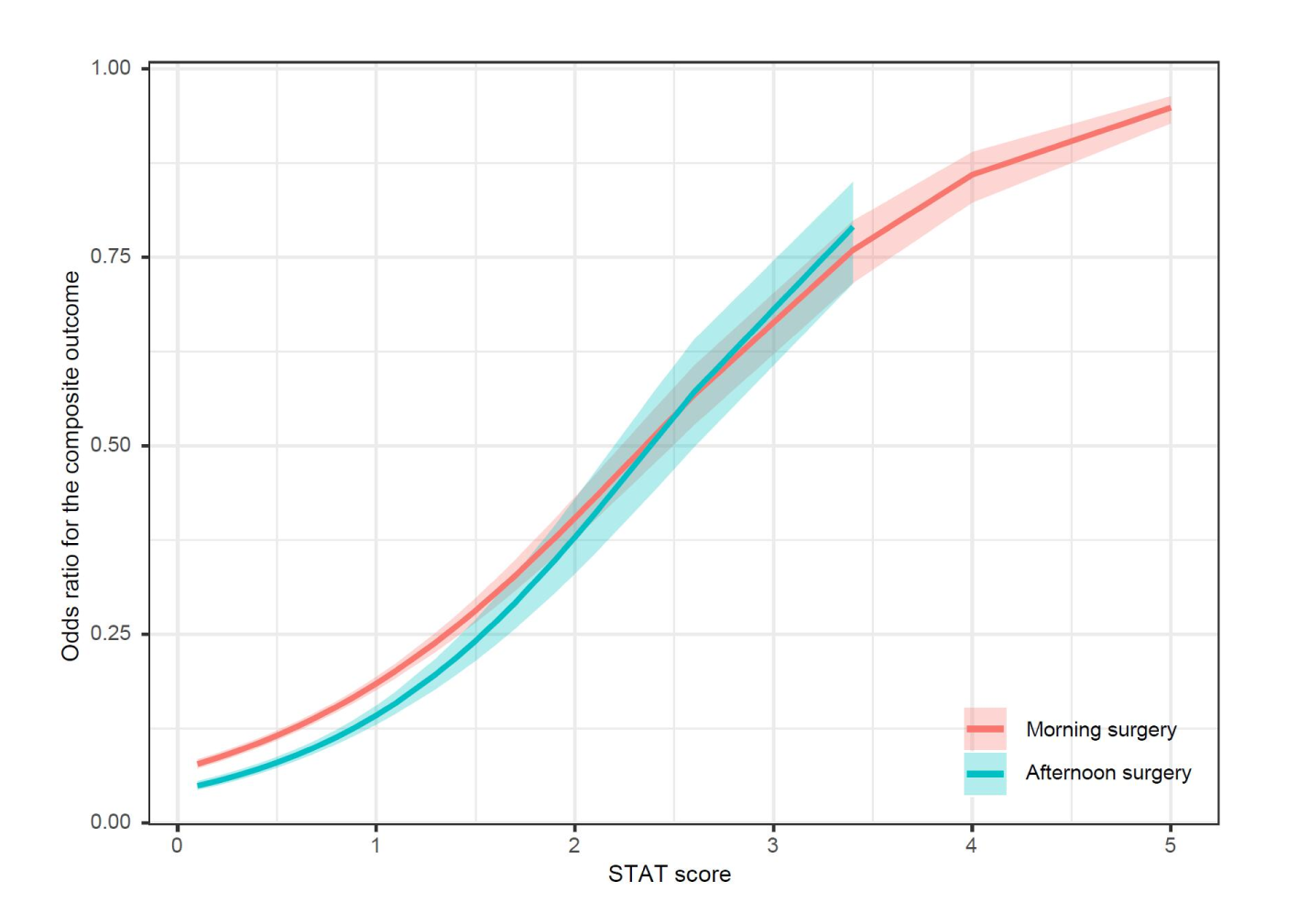

Supplement: Supplemental Material [file IANN_A_2430764_SM8262.zip › suppl_data/Supplementary Figure 2C.tif]
